# Celastrol Alleviates Gamma Irradiation-Induced Damage by Modulating Diverse Inflammatory Mediators

**DOI:** 10.3390/ijms21031084

**Published:** 2020-02-06

**Authors:** Hong Wang, Kwang Seok Ahn, Sulaiman Ali Alharbi, Omar H. M. Shair, Frank Arfuso, Gautam Sethi, Arunachalam Chinnathambi, Feng Ru Tang

**Affiliations:** 1 Department of Pharmacology, Yong Loo Lin School of Medicine, National University of Singapore, Singapore 117600, Singapore; 2 Singapore Nuclear Research and Safety Initiative, National University of Singapore, Singapore 138602, Singapore; 3 College of Korean Medicine, Kyung Hee University, #47, Kyungheedae-gil, Dongdaemoon-gu, Seoul 130-701, Korea; 4 Department of Botany and Microbiology, College of Science, King Saud University, Riyadh 11451, Saudi Arabia; 5 Stem Cell and Cancer Biology Laboratory, School of Pharmacy and Biomedical Sciences, Curtin Health Innovation Research Institute, Curtin University, Perth, WA 6009, Australia

**Keywords:** gamma radiation, celastrol, NF-κB, ROS, in vitro, in vivo

## Abstract

The present study aimed to explore the possible radioprotective effects of celastrol and relevant molecular mechanisms in an in vitro cell and in vivo mouse models exposed to gamma radiation. Human keratinocytes (HaCaT) and foreskin fibroblast (BJ) cells were exposed to gamma radiation of 20 Gy, followed by treatment with celastrol for 24 h. Cell viability, reactive oxygen species (ROS), nitric oxide (NO) and glutathione (GSH) production, lipid peroxidation, DNA damage, inflammatory cytokine levels, and NF-κB pathway activation were examined. The survival rate, levels of interleukin-6 (IL-6) and tumor necrosis factor alpha (TNF-α) in blood, and p65 and phospho-p65 expression were also evaluated in mice after exposure to gamma radiation and celastrol treatment. The gamma irradiation of HaCaT cells induced decreased cell viability, but treatment with celastrol significantly blocked this cytotoxicity. Gamma irradiation also increased free radical production (e.g., ROS and NO), decreased the level of GSH, and enhanced oxidative DNA damage and lipid peroxidation in cells, which were effectively reversed by celastrol treatment. Moreover, inflammatory responses induced by gamma irradiation, as demonstrated by increased levels of IL-6, TNF-α, and IL-1β, were also blocked by celastrol. The increased activity of NF-κB DNA binding following gamma radiation was significantly attenuated after celastrol treatment. In the irradiated mice, treatment with celastrol significantly improved overall survival rate, reduced the excessive inflammatory responses, and decreased NF-κB activity. As a NF-κB pathway blocker and antioxidant, celastrol may represent a promising pharmacological agent with protective effects against gamma irradiation-induced injury.

## 1. Introduction

A gamma ray is a kind of high-frequency electromagnetic radiation consisting of high-energy photons that frees electrons from molecules and ionizes them. It can penetrate the body and cause damage directly at a cellular level, or indirectly on water molecules, which then generate free radicals [1,2]. Free radicals further result in oxidation, DNA damage, and inflammation in the body [1,3,4]. Currently, the probability of radiation exposure in human beings is increased due to the wide use of nuclear energy in modern society, which makes the development of effective radioprotective agents an urgent task [5,6,7]. 

Celastrol, also known as tripterine, is a pentacyclic triterpenoid that belongs to the family of quinone methides [8,9,10,11,12,13,14,15]. It is a natural compound extracted from the root of Chinese medicinal plants *Tripterygium wilfordii* (Thunder god vine) and *Celastrus regelii*. *Tripterygium wilfordii* has demonstrated a significant potential for the treatment of rheumatoid arthritis [16,17,18,19,20], psoriasis vulgaris [21], idiopathic membranous nephropathy and nephrotic syndrome [22,23], diabetic kidney disease [24,25], and Crohn’s disease [26,27]. Meanwhile, the efficacy and safety of celastrol has also been tested in clinical samples. Pinna et al. demonstrated that celastrol inhibited proinflammatory cytokine secretion in Crohn’s disease biopsies from patients [28]. Fang et al. showed that the treatment of celastrol attenuated both the proliferation and invasion of fibroblast-like synoviocytes from patients with rheumatoid arthritis [29]. More importantly, a randomized, placebo-controlled, and double blinded trial showed the potential role of celastrol serving as an effective and safe adjuvant to nifedipine against hypertension in patients with preeclampsia [30]. The functions of celastrol have also been tested in the treatment of neurodegenerative diseases such as Alzheimer’s disease [31,32], the inhibiton of dengue virus replication [33], the protection against insulin resistance induced by mitochondrial dysfunction in human skeletal muscle cells [34,35], the controlling of obesity [36], the prevention of cancer cell proliferation [8,9,10,15,37], and even insecticidal activities [38]. Although the underlying molecular mechanisms are not clear, various biological properties of celastrol, including being an antioxidant [32], anti-inflammatory [39,40], and a modulator of the NF-κB signaling pathway [41], have been observed in both in vitro and in vivo experiments. 

Celastrol was demonstrated to decrease interlukein-6 (IL-6) secretion and gene expression via downregulation of NF-κB in prostate carcinoma PC-3 cells [42], and inhibit colorectal cancer cell growth and migration through the suppression of tumor necrosis factor alpha (TNF-α) expression and IL-1b protein [43]. The gene expression and protein level of IL-6 and TNF-α were also observed to be significantly lowered by celastrol pretreatment in human nucleus pulposus cells [44]. In addition to the above anti-inflammatory effects, celastrol was demonstrated to be a potent NF-κB blocker in attenuating hepatic dysfunction [45], protecting against diabetic liver injury [46], attenuating renal injury [34], potentiating apoptosis, and suppressing tumor cell invasion [15]. As a crucial transcription factor involved in inflammation and oncogenesis, NF-κB can be activated following a variety of stimuli, including radiation-induced oxidative stress [11,47,48,49,50,51,52,53,54]. The NF-κB signaling pathway has been demonstrated to be involved in DNA damage, nitric oxide (NO) production, and the activation of the inflammatory cascade induced by radiation [47,48,55,56,57,58]. Treatment with baicalein [59], brazilin [60], Corilagin [61], and Naringin [48] has been proven to block DNA oxidative damage and inflammation by modulating NF-κB signaling pathway. Therefore, it is hypothesized that celastrol may also exhibit protective effects against gamma radiation-induced injury through regulating NF-κB activity. This study aims to explore the radioprotective effects of celastrol and relevant molecular mechanisms in an in vitro cell and in vivo mouse models exposed to gamma radiation.

## 2. Results

### 2.1. Celastrol Dose-Dependently Reversed Gamma Irradiation-Induced Decrease in Cell Viability

**Figure 1:**
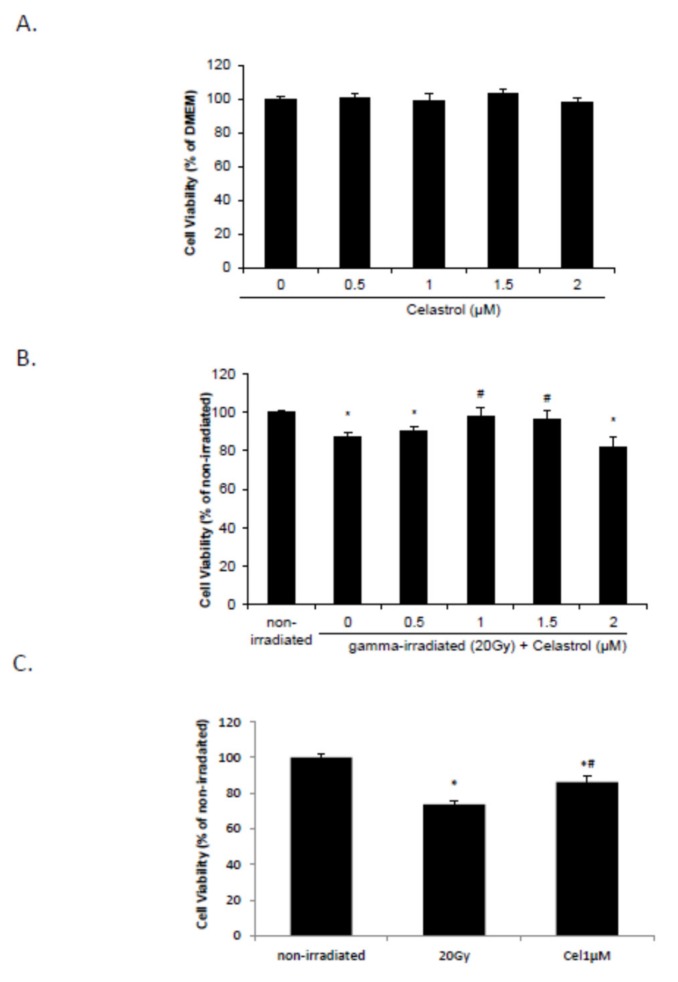
Cell viability after celastrol treatment in HaCaT cells and human skin fibroblast cells. (**A**) HaCaT cells were treated with celastrol at various concentrations of 0, 0.5, 1, 1.5, and 2 µM without gamma radiation. Cell viability was evaluated by MTT after 24 h. (**B**) HaCaT cells were subjected to gamma irradiation with 20 Gy followed by 24 h treatment with celastrol at various concentrations, and cell viability was evaluated by MTT. Dulbecco’s modified Eagle medium (DMEM) group without any drug treatment and gamma radiation served as control. (**C**) Human skin fibroblast cells were subjected to gamma irradiation with 20 Gy, followed by celastrol treatment at 1µM. Cell viability was evaluated by MTT 24 h post-exposure. Eagle’s Minimum Essential Medium (EMEM) group without any drug treatment and gamma radiation served as control. All data are expressed as mean ± S.E.M (*n* = 5). Comparisons between groups were analyzed by One-Way ANOVA followed by Tukey’s post-hoc test. * *p* < 0.05 vs. non-irradiated. # *p* < 0.05 vs. irradiated and no drug treatment.

Our previous study has demonstrated that gamma irradiation dose- (10 to 40 Gy) and time- (24 to 96 h) dependently decreased cell viability in HaCaT cells [62]. Twenty-four hours post-treatment with 1 and 1.5 µM celastrol after irradiation with 20 Gy significantly reversed the irradiation-induced cell viability decrease (Figure 1B), while treatment with 0.5 to 2 µM celastrol did not influence cell viability in HaCaT cells without radiation exposure (Figure 1A). Exposure to 20 Gy gamma radiation also significantly decreased the cell viability in BJ human skin fibroblast cells at 24 h, but 1 µM celastrol treatment reversed this decrease (Figure 1C).

### 2.2. Celastrol Blocked the Increased Reactive Oxygen Species (ROS) and NO Production and Decreased Glutathione (GSH) Levels Induced by Gamma Irradiation

**Figure 2:**
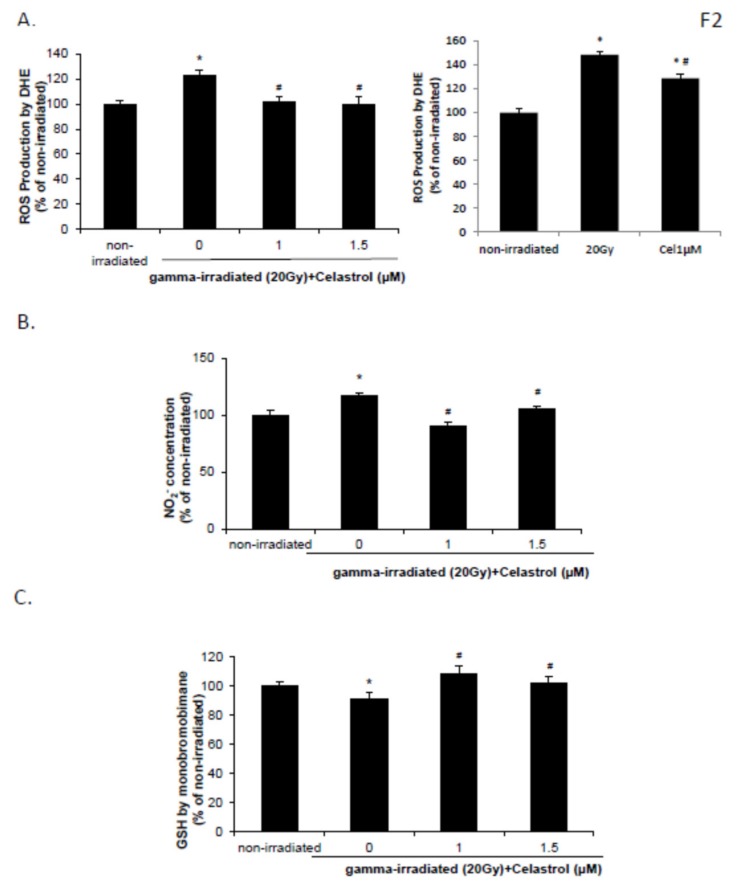
Effects of celastrol on free radical and glutathione (GSH) production in HaCaT and human skin fibroblast cells after gamma irradiation. HaCaT and human skin fibroblast cells were subjected to gamma irradiation with 20 Gy followed by 24 h treatment with celastrol at designated concentrations. DMEM or EMEM group without any drug treatment and gamma radiation exposure served as the control. (**A**) Reactive oxygen species (ROS) production in HaCaT (left panel) and skin fibroblast cells (right panel) was assayed by DHE. (**B**) NO_2_^−^ concentration was evaluated in HaCaT cells by Griess reagent. (**C**) GSH production was evaluated in HaCaT cells. All data are expressed as mean ± S.E.M (*n* = 5). Comparisons between groups were analyzed by One-Way ANOVA followed by Tukey’s post-hoc test. * *p* < 0.05 vs. non-irradiated. # *p* < 0.05 vs. irradiated and no drug treatment.

The DHE assay demonstrated that ROS production in HaCaT cells was increased at 24 h following exposure to 20 Gy of gamma radiation, but 1 and 1.5 µM celastrol treatment significantly reversed this increase and decreased the ROS production (Figure 2A, left panel). Irradiation with 20 Gy also increased the NO level and decreased the GSH level in HaCaT cells (Figure 2B,C). The effects were reversed by treatment with 1 and 1.5 µM celastrol (Figure 2B,C), suggesting potent antioxidative activities of celastrol in HaCaT cells. BJ human skin fibroblast cells exhibited similar results, with enhanced ROS production after 20 Gy gamma radiation but a significant decrease following celastrol treatment (Figure 2A, right panel).

### 2.3. Celastrol Reversed the Increased Lipid Peroxidation and DNA Oxidative Damage Induced by Gamma Irradiation

**Figure 3:**
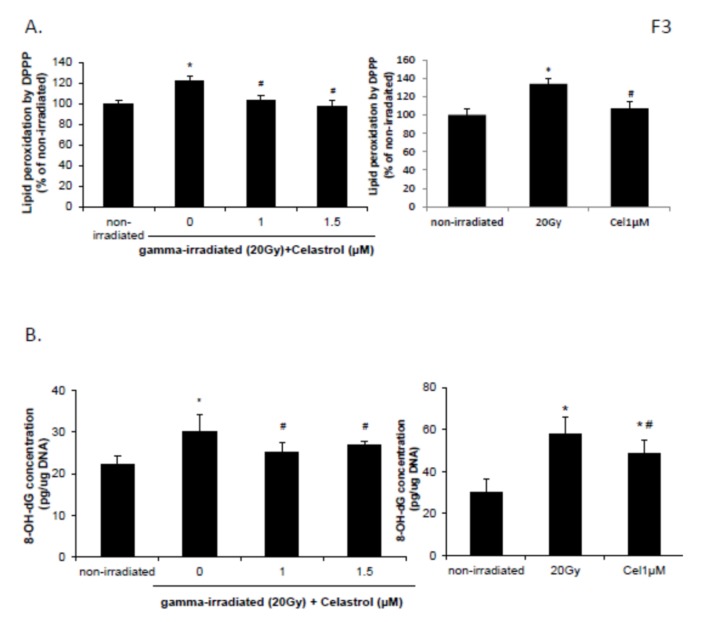
Effects of celastrol on lipid peroxidation (**A**) and oxidative DNA damage (**B**) in HaCaT cells (left panel) and skin fibroblast cells (right panel) after gamma irradiation. Cells were subjected to gamma irradiation with 20 Gy followed by 24 h treatment with celastrol at designated concentrations. DMEM or EMEM group without any drug treatment and gamma radiation exposure served as control. (**A**) Lipid peroxidation was assayed by DPPP. (**B**) Oxidative DNA damage was evaluated by 8-OH-dG EIA kit. All data are expressed as mean ± S.E.M (*n* = 5). Comparisons between groups were analyzed by One-Way ANOVA followed by Tukey’s post-hoc test. * *p <* 0.05 vs. non-irradiated. # *p <* 0.05 vs. irradiated and no drug treatment.

HaCaT cells demonstrated increased lipid peroxidation at 24 h following exposure to 20 Gy of gamma radiation (Figure 3A, left panel). Both 1 and 1.5 µM celastrol treatment significantly reversed this increase (Figure 3A). Enhanced DNA damage induced by gamma radiation was also significantly blocked with the treatment of 1 and 1.5 µM celastrol in HaCaT cells (Figure 3B, left panel). This suggests that the radioprotection of celastrol against gamma radiation-induced injury in HaCaT cells was through inhibiting the oxidative stress cascade. BJ human skin fibroblast cells showed similar effects. The increased DNA oxidative damage and lipid peroxidation following 20 Gy gamma radiation were significantly reversed with 1µM celastrol treatment (Figure 3A, right panel and B, right panel).

### 2.4. Celastrol Blocked the Increased Levels of proInflammation Cytokines and the Activation of NF-κB Pathway Induced by Gamma Irradiation

**Figure 4:**
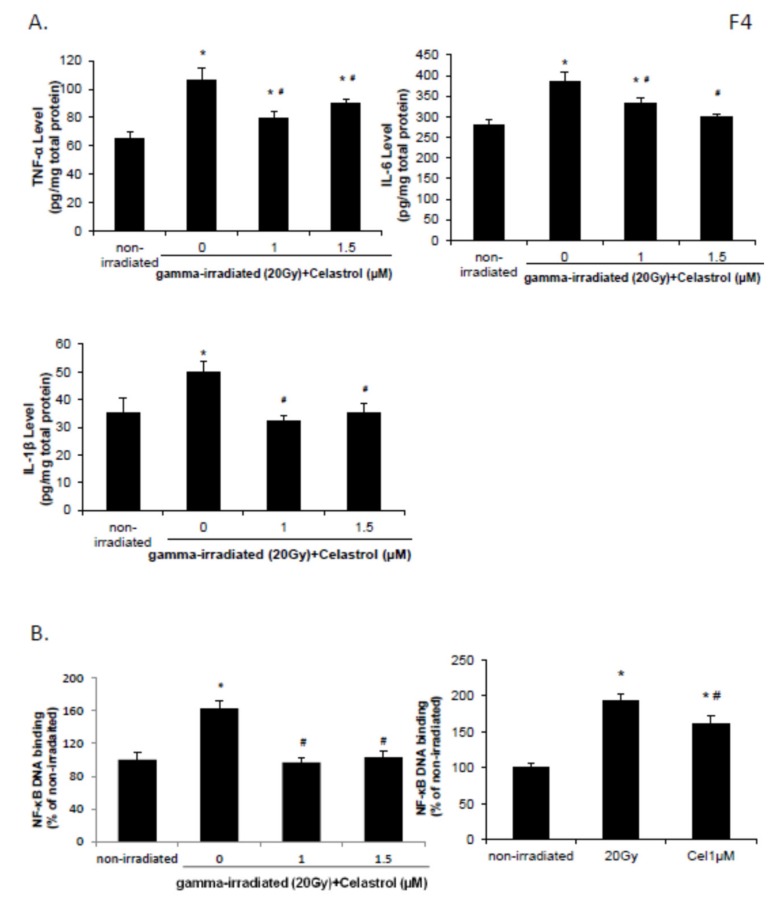
Effects of celastrol on the levels of inflammatory cytokines and NF-κB activation. Cells were subjected to gamma irradiation with 20 Gy, followed by 24 h treatment with celastrol at designated concentrations. DMEM or EMEM group without any drug treatment and gamma radiation exposure served as control. (**A**) The levels of tumor necrosis factor alpha (TNF-α), interleukin-6 (IL-6), and IL-1β in HaCaT cell lysate were evaluated using ELISA. (**B**) NF-κB DNA binding assay was performed 24 h post gamma-radiation in HaCaT (left panel) and human skin fibroblasts (right panel). All data are expressed as mean ± S.E.M (*n* = 3). Comparisons between groups were analyzed by One-Way ANOVA followed by Tukey’s post-hoc test. * *p <* 0.05 vs. non-irradiated. # *p <* 0.05 vs. irradiated and no drug treatment.

IL-6, TNF-α, and IL-1β levels were increased in HaCaT cells at 24 h following exposure to 20 Gy gamma radiation when compared with nonirradiated control group (Figure 4A). The enhancement was significantly inhibited by treatment with 1 and 1.5 µM celastrol, suggestive of the anti-inflammatory activities of celastrol in HaCaT cells.

Exposure to gamma radiation of 20 Gy also increased the DNA binding activity of NF-κB in HaCaT cells (Figure 4B), indicative of activation of the NF-κB pathway after gamma irradiation. Celastrol treatment at 1 and 1.5 µM significantly reversed this increase (Figure 4B), suggesting the NF-κB inhibition potential of celastrol. DNA binding activity of NF-κB increased following exposure to 20 Gy of gamma radiation in BJ human skin fibroblast cells, and this enhancement was reversed by 1µM celastrol treatment (Figure 4B).

### 2.5. Celastrol Treatment Significantly Increased Mouse Survival Rate Following Gamma Radiation Exposure

**Figure 5:**
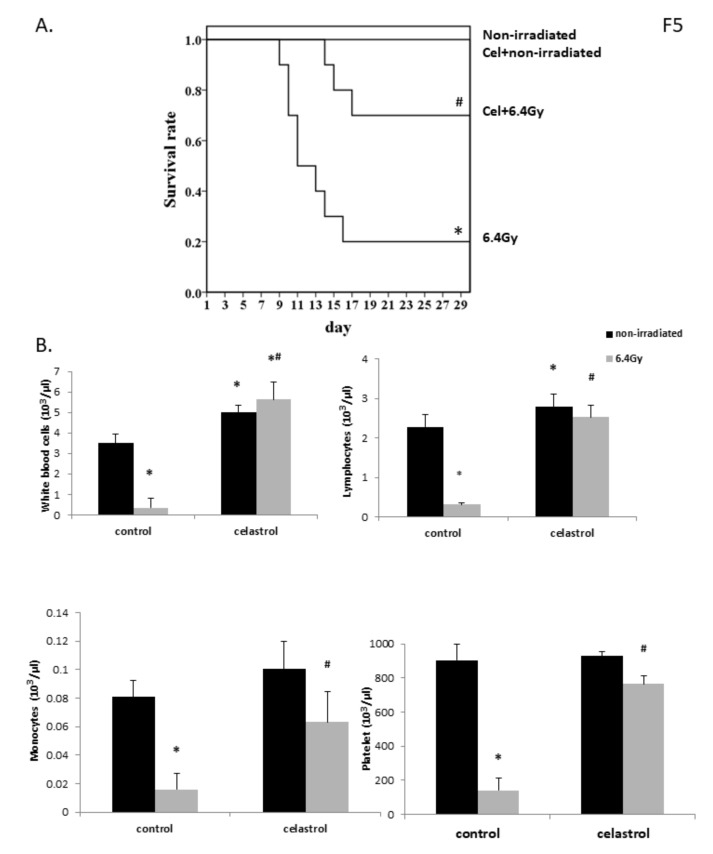
Effects of celastrol treatment on the survival rate (**A**) and hematopoietic level (**B**) in gamma irradiated mice. Four groups of mice were employed. Nonirradiated control and celastrol groups received either ip injection of 0.1% DMSO or celastrol at 0.25 mg/kg bw for 30 days without exposure to gamma irradiation. The irradiated mice were subjected to 6.4 Gy gamma irradiation at day 1, followed by ip injection of 0.1% DMSO or celastrol at 0.25 mg/kg bw for 30 days. (**A**) Kaplan–Meier curve of the effects of celastrol on the survival rate of mice. (**B**) The numbers of white blood cells, lymphocytes, monocytes, and platelets. Data are expressed as mean ± S.E.M (*n* = 3 for 6.4 Gy group and *n* = 5 for other groups). Comparisons between groups were analyzed by One-Way ANOVA followed by Tukey’s post-hoc test. * *p <* 0.05 vs. non-irradiated and no drug treatment group. # *p <* 0.05 vs. irradiated and no drug treatment group.

Mice from nonirradiated control and nonirradiated celastrol groups exhibited no death after 30 days with saline or celastrol treatment. Gamma-irradiation with 6.4 Gy induced 80% animal death at 30 days, while celastrol treatment at 0.25 mg/kg body weight (bw) in the mice significantly enhanced the survival rate to 70% (Figure 5A), indicating that the treatment with celastrol effectively protected against the damage induced by gamma irradiation in mice.

### 2.6. Celastrol Treatment Exerted Protective Effects in Mice Against Myelosuppression Induced by Gamma Irradiation

Exposure to 6.4 Gy gamma irradiation promoted the development of myelosuppression: leucopenia, monocytopenia, lymphocytopenia, and thrombocytopenia in mice, demonstrated by the decreased numbers of white blood cells, monocytes, lymphocytes, and platelets in mouse blood (Figure 5B). The treatment with celastrol for 30 day in mice significantly improved the levels of hematopoietic cells. The nonirradiated celastrol group also showed increased levels of white blood cells and lymphocytes compared with the control mice. These observations demonstrated the function of celastrol in improving myelosuppression in mice at the hematopoietic level.

### 2.7. Celastrol Exhibited Anti-Inflammatory Activities in Animals Following Exposure to Gamma Irradiation

**Figure 6:**
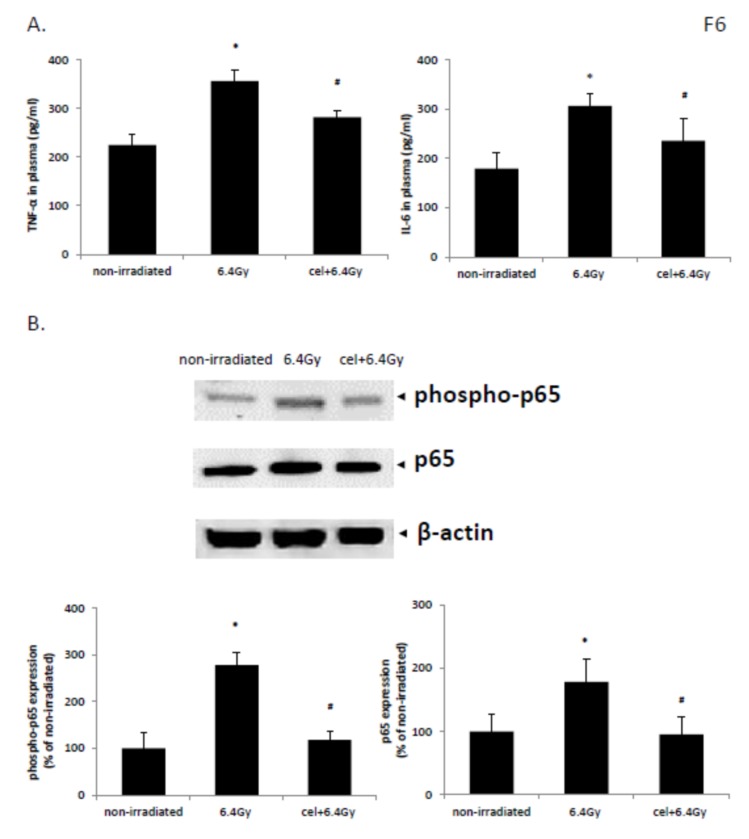
Effects of celastrol treatment on the expression of inflammatory cytokines (**A**) and NF-κB activation (**B**). Nonirradiated control mice received the ip injection of 0.1% DMSO for 30 days without exposure to gamma irradiation. Both 6.4 Gy and cel + 6.4 Gy were subjected to gamma irradiation with 6.4 Gy at day 1, followed by ip injection of 0.1% DMSO or celastrol at 0.25 mg/kg bw for 30 days respectively. (**A**) The expression levels of inflammatory cytokines TNF-α and IL-6 in plasma were evaluated by ELISA (*n* = 3 for 6.4 Gy group and *n* = 5 for other groups). (**B**) Protein expressions of p65, phospho-p65, and β-actin were evaluated by Western blot analysis in the colon tissue of mice. The results were calculated and expressed as fold change relative to control (*n* = 3). Data are expressed as mean ± S.E.M. Comparisons between groups were analyzed by One-Way ANOVA followed by Tukey’s post-hoc test. * *p <* 0.05 vs. non-irradiated. # *p <* 0.05 vs. irradiated and no drug treatment.

Exposure to gamma irradiation of 6.4 Gy significantly increased the concentration of inflammatory cytokines TNF-α and IL-6 in mouse plasma when compared with those from nonirradiated control mice (Figure 6A). Treatment with celastrol significantly downregulated the expression levels of TNF-α and IL-6 in plasma, indicating anti-inflammatory activities of celastrol after exposure to gamma-irradiation in mice.

### 2.8. Celastrol Reversed NF-κB Pathway Activation Induced by Gamma Irradiation in Mice

The protein level of p65 and phospho-p65 was enhanced in the animal colon tissues following exposure to 6.4 Gy gamma irradiation, indicating NF-κB pathway activation. This increase was reversed upon celastrol treatment (Figure 6B). These results demonstrated that celastrol acts as an inhibitor of the NF-κB pathway in reducing the activity and phosphorylation of NF-κB.

## 3. Discussion

The possibility of humans being exposed to gamma radiation is increased due to the use of radiation for medical diagnosis or treatment and the generation of nuclear power. This has made the development of effective and safe radioprotectors an important issue. The discovery of compounds with fewer toxic effects isolated from natural sources has enabled the identification of new potential radioprotective agents [63,64]. A traditional Chinese herb, Dragon’s blood, and its extracts were found to show antioxidative and anti-inflammatory activities and effectively alleviate radiation-induced damage in bone marrow [65]. Treatment with Ligustrazine, a key component of the Chinese herb Chuanxiong, decreased mortality in mice after whole-body γ-irradiation through its antioxidative function in reducing ROS levels, DNA damage, and apoptosis as well as activating survival signal Akt pathways [66]. Polysaccharides, which are the most important functional constituents in *Lycium barbarum* fruits, showed significant protective effects on the reproductive system of male rats impaired by local exposure to gamma irradiation [67]. *Podophyllum hexandrum*, also known as the Himalayan May Apple, showed radioprotective effects in lethally irradiated mice [68,69]. Its extracts exhibited antioxidative potential in inhibiting nitric oxide production and promoting DNA repair [70,71]. REC-2006, a bioactive fractionated extract from the rhizome of *Podophyllum hexandrum*, prevented radiation-induced DNA damage and stimulated its repair in murine thymocytes in vivo [72]. *Acorus calamus*, from the Acoraceae family, was found to exert radioprotective effects in mouse liver homogenates [73] or in mice that received whole-body gamma irradiation [74]. These studies highlight that extracts from natural products may represent potential protective agents against radiation-induced injuries.

In this study, we employed celastrol, a pentacyclic triterpenoid from the quinone methides family, and evaluated its potential protective activities in alleviating gamma radiation-induced cell and tissue injury. It was found that treatment with celastrol from 0.5 to 2 µM showed no toxicity in HaCaT cells. Our in vivo study also demonstrated that mice with daily administration of 0.25 mg/kg bw celastrol for 30 days exhibited no adverse effects. So far, very few studies have been published regarding the protective effect of celastrol against the damage induced by gamma radiation, although its functions in inhibiting the proliferation of cancer cells are well recognized [8,9,10,15]. This is the first study to explore the effect of celastrol on the injury induced by gamma radiation in animals and in keratinocytes and fibroblasts.

It is well known today that oxidative and nitrosative stresses are potent pathogenic mechanisms contributing to gamma radiation-induced damage. Recent studies have proposed that gamma radiation significantly increased intracellular ROS and reactive nitrogen species formation [1,62]. Our results demonstrated that gamma irradiation with 20 Gy significantly decreased cell viability in keratinocytes, accompanied by increased ROS production and NO release and decreased GSH levels. These changes further increased lipid peroxidation and DNA damage levels, which are responsible for the cell death induced by gamma radiation. Some assays were also conducted in the BJ human skin fibroblast cell line in order to confirm our observation in keratinocytes. The inflammatory process is a key consequence after gamma radiation exposure, which is characterized by the increased levels of pro-inflammatory cytokines, including IL-6, TNF-α, and IL-1β [75,76]. Our study detected the enhancement in these inflammatory cytokines in cells and mice after gamma radiation exposure. Inflammation, lipid peroxidation, and oxidative DNA damage are all critical events in the reaction of the free radical chain, which contributes to the various deleterious effects after gamma radiation exposure. Celastrol, as an anti-inflammatory agent and antioxidant, was demonstrated to block gamma irradiation-induced cell death at 1 and 1.5 µM concentrations. The effect was implemented by the decrease in the production of ROS and NO, lipid peroxidation, oxidative DNA damage, and inflammatory response in human keratinocytes and fibroblasts. Our results are in agreement with a report that showed the ability of celastrol to reduce DNA damage in human prostate cancer [77]. Another study also elaborated the protective effects of celastrol on human peripheral blood mononuclear cells against radiation-induced oxidative stress through reducing ROS production and increasing antioxidant enzymes such as manganese superoxide dismutase (MnSOD) and catalase [78]. Han et al. recently reported that celastrol treatment not only attenuated gamma radiation-induced cytotoxicity in human umbilical vein endothelial cells, but it also effectively blocked the enhanced oxidative stress through increasing the levels of superoxide dismutase (SOD), catalase, glutathione S-transferase (GST) and glutathione peroxidase (GPx) [79]. All these studies highlight the potential protective effect of celastrol against gamma radiation-induced damage.

NF-κB signaling pathways have been known to be activated by diverse mechanisms after gamma irradiation [80]. The regulation of the NF-κB pathway is involved in the survival and death of cells exposed to gamma radiation [48]. Our previous study indicated that gamma radiation exposure time- and dose-dependently enhanced NF-κB DNA binding activities in HaCaT cells [62], while celastrol treatment was found to suppress this increase. Our observations were in agreement with a recent study [48]. Naringin was proved to inhibit inflammation and oxidative DNA damage induced by gamma radiation through the regulation of p53 and NF-κB signaling pathways in animal splenocytes. In another study, Lee et al. (2014) demonstrated that skin melanoma cells showed a proliferation loss and NF-κB pathway activation after UV light exposure [81]. All the above findings support our hypothesis that the protective effect of celastrol against gamma radiation-induced ROS production, lipid peroxidation, and DNA damage could be mediated through negative regulation of the NF-κB signaling cascade.

The protective effect of celastrol in gamma radiation was also evaluated in vivo in the present study. Oxidative stress, lipid peroxidation, DNA breaks, and p53/ NF-κB activities have been previously studied in animal models after exposure to gamma radiation [82,83]. It has previously been demonstrated that death of mice occurred after ^137^Cs [84] and ^60^Co gamma radiation [82,85,86]. Our study also observed 80% animal death at 30 days after exposure to radiation of 6.4 Gy using ^137^Cs as an irradiating source, and post-treatment with celastrol for 30 days significantly increased the mouse survival rate after whole-body gamma irradiation exposure. One possible mechanism contributing to the radioprotective effect of celastrol might be via alleviating the damage induced in the animals’ hematopoietic system. Hematopoietic syndrome has been shown to be a cause of death in animals exposed to total body gamma irradiation, and it mainly occurs within 30 days post-exposure [87,88,89]. In our study, the numbers of white blood cells, monocytes, lymphocytes, and platelets dropped in mouse plasma after gamma irradiation exposure, indicative of leucopenia, monocytopenia, lymphocytopenia, and thrombocytopenia in the animals. The thirty-day post-treatment with celastrol significantly mitigated this syndrome, as suggested by the enhanced levels of white blood cells, monocytes, lymphocytes, and platelets when compared to the irradiated mice without celastrol treatment. These data suggest that celastrol treatment can result in the restoration of the hematopoietic system. A slight increase in the number of lymphocytes and white blood cells was also observed in animals without gamma irradiation after celastrol treatment. Lymphocytes contain different cell types (e.g., B cells and T cells), and the levels change according to gender, drug treatment, disease, and so forth. So far, no report has been published on the direct function of celastrol on lymphocyte numbers in mice or humans, although evidence showed that celastrol acts as a modulator of the hematopoietic system in mice [90]. Several studies pointed out the role of celastrol in altering the balance between T helper 17 (Th17) and regulatory T cells (Treg) by suppressing Th17 cell induction and promoting the generation of Treg cells [91,92,93]. Celastrol also increased Th2 cells while decreased Th1 cells accompanied by a significant reduction in NF-κB expression in multiple sclerosis in rats [94]. In order to clarify the exact role of celastrol in the hematopoietic system, our future work may examine the individual cell types of lymphocytes after celastrol treatment in mice.

Another possible mechanism contributing to the radioprotective effects of celastrol was its ability to attenuate the inflammatory reactions in animals after gamma radiation exposure. The circulating concentrations of two inflammatory cytokines, TNF-α and IL-6, were increased in the plasma of surviving mice after gamma irradiation, while treatment with celastrol significantly lowered the levels. This result is supported indirectly by a few studies. One showed that the increased levels of circulating TNF-α and IL-6 in mice exposed to gamma radiation were attenuated by ferulic acid treatment [82]. Another study demonstrated the increased IL-6 and TNF-α expression and lipid peroxidation in mice after gamma radiation exposure, accompanied by NF-κB pathway activation [95]. It is well accepted that gamma radiation is associated with ROS generation and the activation of redox-sensitive transcription factor NF-κB [96]. NF-κB further promoted the inflammatory responses by controlling the levels of inflammatory cytokines (e.g., IL-6 and TNF-α). Our study showed that treatment with celastrol suppressed gamma irradiation-induced enhancement of p65 and phospho-p65 expression. It was found that some antioxidants such as ferulic acid and epicatechin showed similar mechanisms as celastrol, in terms of reducing NF-κB activity and oxidative stress in gamma-irradiated mice [82,95].

In summary, celastrol, functioning as a potent NF-κB pathway inhibitor and an antioxidant, showed protective effects against gamma radiation-induced damage. It decreased cell death, ROS and NO production, lipid peroxidation, DNA oxidative damage, and inflammatory responses via suppressing the NF-κB pathway. Moreover, treatment with celastrol improved survival rate in mice after gamma irradiation exposure, reduced excessive inflammatory responses, protected the hematopoietic system, and reversed the increased levels of phospho-p65 and p65 induced by gamma irradiation. Given its pharmaceutical properties and radioprotective efficacy, celastrol may act as a potential radioprotective agent against the deleterious effects induced by gamma irradiation.

## 4. Materials and Methods

### 4.1. Reagents

Celastrol, nuclease P1, alkaline phosphatase, dihydroethidium (DHE), dimethyl sulfoxide (DMSO), sodium dodecyl sulfate (SDS), Tris, glycine, bovine serum albumin (BSA), 3-(4,5-dimethylthiazol-2-yl)-2,5-diphenyltetrazolium bromide (MTT), and CelLytic^TM^ mammalian tissue lysis/extraction reagent were from Sigma-Aldrich (St. Louis, MO, USA). Monobromobimane (mBBr) was obtained from Thermo Fisher Scientific (Waltham, MA, USA). Dulbecco’s modified Eagle medium (DMEM), fetal bovine serum (FBS), and antibiotic/antimycotic mixtures were purchased from Invitrogen (Carlsbad, CA, USA). Purification kits for genomic DNA and Griess reagent were from Promega (Madison, WI, USA). Diphenyl-1-pyrenylphosphine (DPPP) and 8-hydroxy-2-deoxy Guanosine (8-OH-dG) EIA kits were purchase from Cayman Chemical Company (Ann Arbor, MI, USA). Kits for nuclear extraction and NF-κB DNA binding were purchased from Active Motif (CA, USA). ELISA kits for tumor necrosis factor alpha (TNF-α), interleukin-6 (IL-6), and interleukin-1beta (IL-1β) were from R&D systems (Minneapolis, MN, USA). Antibodies against β-actin, p65, and phospho-specific p65 (Ser 536) were from Cell Signaling Technology (Beverly, MA, USA). Horseradish peroxidase (HRP)-conjugated goat anti-mouse and anti-rabbit antibodies were purchased from Santa Cruz Biotechnology (Santa Cruz, CA, USA). Nitrocellulose membranes and Bradford reagents were purchased from Bio-Rad (Hercules, CA, USA). ECL Reagent for Western blotting was obtained from GE healthcare (Buckinghamshire, UK).

### 4.2. Cell Lines

HaCaT and BJ cells were obtained and maintained as previously described [62]. HaCaT cells were provided by Dr. Francoise Thierry, Institute of Medical Biology, Agency for Science Technology and Research (A*STAR), Singapore. HaCaT cells are in vitro, spontaneously transformed immortalized keratinocytes from histologically normal skin. These cells were cultured in DMEM containing 10% FBS and 1% antibiotic/antimycotic solution. BJ human skin fibroblast cells were purchased from ATCC (CRL-2522, Manassas, VA, USA) and cultured in ATCC-formulated Eagle’s Minimum Essential Medium (EMEM) containing 10% FBS and 1% antibiotic-antimycotic solution. All cells were maintained at 37 °C in a humidified 5% CO_2_ atmosphere.

### 4.3. Irradiation Procedure and Drug Treatment

BIOBEAM 8000 using Cesium-137 as a radioactive source (Gamma-Service Medical GmbH, Leipzig, Germany) was used in this study. Cells were exposed to gamma radiation with indicated doses at a dose rate of 2.6 Gy/min and then treated with celastrol at designated concentrations for 24 h. Celastrol at 100 mM in DMSO was kept at 4 °C as a stock solution. The cells were collected at designated time points post-irradiation for various assays. The nonirradiated DMEM or EMEM group was used as the control.

### 4.4. Cell Viability Assay

The 3-(4,5-dimethylthiazol-2-yl)-2,5-diphenyltetrazolium bromide (MTT) method was used to evaluate cell viability after irradiation and celastrol treatment. Briefly, 100 μL cells were incubated in a 96-well plate with or without drug treatment. Twenty microliters of 5 mg/mL MTT in PBS was added to the plate and incubated at 37 °C. After 4 h, the supernatant was removed, and 100 µL DMSO was added and incubated at 37 °C for 1 h. A Tecan Safire microplate reader (Thermo Fisher Scientific, Waltham, MA, USA) was used to measure optical density (OD) at 570 nm.

### 4.5. Reactive Oxygen Species (ROS), NO, and Glutathione (GSH) Assays

Dihydroethidium (DHE) was employed to measure ROS production. Cells were treated with 5 µM DHE for 30 min at 37 °C in the dark. The fluorescence intensity was measured using a Varioskan Flash microplate reader (Thermo Scientific, Waltham, MA, USA) at the excitation and emission wavelengths of 535 and 610 nm.

The concentration of NO_2_^−^ was assayed by Griess reagent. A 50 µL sample or nitrite standard, 50 µL N-1-naphthylethylenediamine dihydrochloride (NED) Solution, and 50 µL sulfanilamide solution were mixed and incubated for 10 min at room temperature in the dark. The absorbance of the formed purple/magenta color at 520 nm was measured within 30 min using a Tecan Safire microplate reader (Thermo Fisher Scientific, Waltham, MA, USA). GSH levels were assayed by monobromobiman (mBBr), which turns from a nonfluorescent to fluorescent form when conjugated with low molecular weight thiols (e.g., glutathione). Cells were treated with 2mM mBBr at 37 °C for 30 min in the dark, followed by the fluorescent reading using a Varioskan Flash microplate reader (Thermo Scientific, Waltham, MA, USA). The glutathione conjugate of mBBr has absorption/emission spectra at about 394/490 nm.

### 4.6. Lipid Peroxidation Assay

Diphenyl-1-pyrenylphosphine (DPPP) was used to evaluate lipid peroxidation. It yields the fluorescent diphenyl-1-pyrenylphosphine oxide (DPPP-O) upon stoichiometric reaction with hydroperoxides. HaCaT cells were incubated with 50 µM DPPP for 60 min at 37 °C in the dark. The fluorescence was measured using a Varioskan Flash microplate reader (Thermo Scientific, Waltham, MA, USA) at excitation wavelength of 351 nm and emission wavelength of 380 nm, respectively.

### 4.7. DNA Oxidative Damage Assay

An EIA kit for 8-OH-dG detection was used to evaluate DNA damage as previously described [62]. Briefly, cells were harvested by trypsinization. DNA was isolated and incubated with nuclease P1 and alkaline phosphatase at 37 °C for 30 min. A total of 50 µL sample or standard, 50 µL antibodies, and 50 µL tracer were added into the testing plate. After overnight incubation at 4 °C, the plate was washed and incubated with 200 µL Ellman’s reagent for 90–120 min in the dark for optimum development. The absorbance was measured at a wavelength of 420 nm using a Tecan Safire microplate reader (Thermo Fisher Scientific, Waltham, MA, USA). The concentration of 8-OH-dG was calculated according to the standard curve.

### 4.8. Enzyme-Linked Immunosorbent Assay (ELISA)

The levels of IL-6, TNF-α, and IL-1β were evaluated in cell lysates using ELISA. A 100 µL sample or standard was added into a 96-well plate coated by capture antibody. The plate was then incubated at room temperature for 2 h and blocked by reagent diluent. The plate was washed and then incubated with respective detection antibodies, followed by streptavidin-HRP and substrate. The absorption of each well was measured at 450 nm using a Tecan Safire microplate reader (Thermo Fisher Scientific, Waltham, MA, USA). The levels of IL-6, TNF-α, and IL-1β were calculated according to the standard curve.

### 4.9. Extraction of Nuclear and Cytoplasmic Fraction

This procedure was performed as described previously [62]. Cells were washed and collected in 1 x hypotonic buffer. Twenty-five microliters of detergent was added, and the cytoplasmic fraction supernatant was collected after centrifugation at 14,000× *g*. Complete lysis buffer was used to re-suspend the nuclear pellet. After vortexing and centrifugation, the nuclear fraction supernatant was collected and stored at −80 °C.

### 4.10. NF-κB DNA Binding Activity Assay

This procedure was performed as described previously [62]. A 20 µL sample and 30 µL complete binding buffer were incubated at room temperature for 1 h. After washing, the plate was added sequentially with 100 µL NF-κB antibody, HRP-conjugated antibody, and developing solution. The absorbance was measured at a wavelength of 450 nm using a Tecan Safire microplate reader (Thermo Fisher Scientific, Waltham, MA, USA).

### 4.11. In Vivo Irradiation Procedure in Mice

Seven to eight week old Balb/c female mice were obtained from InVivos, Singapore. Animals were housed in the Comparative Medicine Facility, National University of Singapore, with free access to water and food. The protocol was approved by the Institutional Animal Use and Care Committee, National University of Singapore (Institutional Animal Use and Care Committee (IACUC) Number: 081/12). All the in vivo experiments and procedures were performed in accordance with relevant regulations and guidelines specified by IACUC. Forty mice were randomly divided into four groups: nonirradiated control, nonirradiated celastrol, 6.4 Gy, and celastrol + 6.4 Gy. Nonirradiated control and nonirradiated celastrol mice were intraperitoneally injected with 0.1% DMSO or 0.25 mg/kg body weight (bw) celastrol for 30 days without exposure to gamma irradiation. Mice in the 6.4 Gy and cel + 6.4 Gy groups were exposed to 6.4 Gy gamma irradiation at day 1, followed by intraperitoneal injection of 0.1% DMSO or celastrol at 0.25 mg/kg bw for 30 day. The animals’ general health was monitored daily. After animals were euthanized at day 30, blood and colon tissue were collected.

### 4.12. Whole Blood Count

The blood was collected from each surviving animal by heart puncture at day 30 in EDTA tubes. The complete blood count was performed, and the numbers of blood cells (white blood cells, monocytes, and lymphocytes) and platelets were compared.

### 4.13. TNF-α and IL-6 Assayed by ELISA in Animal Plasma

Plasma was collected by centrifugation of blood at 2000× *g* for 10 min. The level of TNF-α and IL-6 in animal plasma was measured using ELISA kits as described above.

### 4.14. Western Blotting for Phospho-p65 and p65 in Mouse Colon Tissues

Protein isolation from mouse colon tissues and Western blotting were performed as described previously [62]. Briefly, cell lysates were loaded on a 10% SDS-PAGE gel and then transferred to a nitrocellulose membrane. The membrane was blocked by 5% BSA and incubated with the respective primary antibodies (β-actin, 1:1000 dilution; p65 and phospho-p65, 1:500 dilution) overnight at 4 °C. After washing, the membrane was then incubated with the respective HRP-conjugated secondary antibodies at room temperature for 1 h. The ECL method was used to visualize the membrane. The images were captured and analyzed using the Bio-Rad Laboratories Gel Doc system. The results were calculated and expressed as fold change relative to control.

### 4.15. Statistical Analysis

Data in a normal distribution were expressed as mean ± S.E.M. One-Way ANOVA and Tukey’s post-hoc tests were employed to compare the between-group differences. The survival rate in the four groups of mice was analyzed by the Kaplan–Meier method. Statistical significance was set as *p* value < 0.05.

## Abbreviations

ROS	reactive oxygen species
MTT	3-(4,5-dimethylthiazol-2-yl)-2,5-diphenyltetrazolium bromide
DMSO	dimethyl sulfoxide
DHE	dihydroethidium
SDS	sodium dodecyl sulphate
BSA	bovine serum albumin
DMEM	Dulbecco’s Modified Eagle Medium
FBS	fetal bovine serum
DPPP	diphenyl-1-pyrenylphosphine
DPPP-O	diphenyl-1-pyrenylphosphine oxide
GSH	glutathione
mBBr	monobromobimane
8-OH-dG	8-hydroxy-2-deoxy Guanosine
IL-6	interleukin-6
TNF-α	tumor necrosis factor alpha
IL-1β	interleukin-1beta
HRP	horseradish peroxidase
ELISA	enzyme-linked immunosorbent assay
ECL	chemiluminescence
IACUC	Institutional Animal Use and Care Committee
DIRP	Defence Innovative Research Programme

## References

[1] Wang H., Sethi G., Loke W.K., Sim M.K. (2015). Des-Aspartate-Angiotensin I Attenuates Mortality of Mice Exposed to Gamma Radiation via a Novel Mechanism of Action. PLoS ONE.

[2] Lee Y.H., Kumar N.C., Glickman R.D. (2012). Modulation of photochemical damage in normal and malignant cells by naturally occurring compounds. Photochem. Photobiol..

[3] Fernandez-Viadero C., Jimenez-Sanz M., Fernandez-Perez A., Verduga Velez R., Crespo Santiago D. (2016). Inflammation and oxidation: Predictive and/or causative factors. Rev. Esp. Geriatr. Gerontol..

[4] Haase G.M., Prasad K.N. (2016). Oxidative Damage and Inflammation Biomarkers: Strategy in Hearing Disorders. Otol. Neurotol..

[5] Fesenko S., Balonov M., Prohl G., Nakayama S., Howard B.J. (2017). A Comparison of Remediation after the Chernobyl and Fukushima Daiichi Accidents. Radiat. Prot. Dosim..

[6] Ikenoue T., Takata H., Kusakabe M., Kudo N., Hasegawa K., Ishimaru T. (2017). Temporal variation of cesium isotope concentrations and atom ratios in zooplankton in the Pacific off the east coast of Japan. Sci. Rep..

[7] Thompson M.A. (2001). Maintaining a proper perspective of risk associated with radiation exposure. J. Nucl. Med. Technol..

[8] Kannaiyan R., Hay H.S., Rajendran P., Li F., Shanmugam M.K., Vali S., Abbasi T., Kapoor S., Sharma A., Kumar A.P. (2011). Celastrol inhibits proliferation and induces chemosensitization through down-regulation of NF-kappaB and STAT3 regulated gene products in multiple myeloma cells. Br. J. Pharmacol..

[9] Kannaiyan R., Manu K.A., Chen L., Li F., Rajendran P., Subramaniam A., Lam P., Kumar A.P., Sethi G. (2011). Celastrol inhibits tumor cell proliferation and promotes apoptosis through the activation of c-Jun N-terminal kinase and suppression of PI3 K/Akt signaling pathways. Apoptosis.

[10] Kannaiyan R., Shanmugam M.K., Sethi G. (2011). Molecular targets of celastrol derived from Thunder of God Vine: Potential role in the treatment of inflammatory disorders and cancer. Cancer Lett..

[11] Shanmugam M.K., Ahn K.S., Lee J.H., Kannaiyan R., Mustafa N., Manu K.A., Siveen K.S., Sethi G., Chng W.J., Kumar A.P. (2018). Celastrol Attenuates the Invasion and Migration and Augments the Anticancer Effects of Bortezomib in a Xenograft Mouse Model of Multiple Myeloma. Front. Pharmacol..

[12] Shanmugam M.K., Warrier S., Kumar A.P., Sethi G., Arfuso F. (2017). Potential Role of Natural Compounds as Anti-Angiogenic Agents in Cancer. Curr. Vasc. Pharmacol..

[13] Rajendran P., Li F., Shanmugam M.K., Kannaiyan R., Goh J.N., Wong K.F., Wang W., Khin E., Tergaonkar V., Kumar A.P. (2012). Celastrol suppresses growth and induces apoptosis of human hepatocellular carcinoma through the modulation of STAT3/JAK2 signaling cascade in vitro and in vivo. Cancer Prev. Res..

[14] Shanmugam M.K., Lee J.H., Chai E.Z., Kanchi M.M., Kar S., Arfuso F., Dharmarajan A., Kumar A.P., Ramar P.S., Looi C.Y. (2016). Cancer prevention and therapy through the modulation of transcription factors by bioactive natural compounds. Semin. Cancer Biol..

[15] Sethi G., Ahn K.S., Pandey M.K., Aggarwal B.B. (2007). Celastrol, a novel triterpene, potentiates TNF-induced apoptosis and suppresses invasion of tumor cells by inhibiting NF-kappaB-regulated gene products and TAK1-mediated NF-kappaB activation. Blood.

[16] He W.Z., Yin Z.H., Gao J.H., Ye Z.Z., Xie Y., Kong W.H., Chen Y.S. (2014). Etanercept combined with Tripterygium wilfordii polyglycoside for treatment of rheumatoid arthritis in the elderly: A clinical study. Zhongguo Zhong Xi Yi Jie He Za Zhi Zhongguo Zhongxiyi Jiehe Zazhi = Chin. J. Integr. Tradit. West. Med..

[17] Lv Q.W., Zhang W., Shi Q., Zheng W.J., Li X., Chen H., Wu Q.J., Jiang W.L., Li H.B., Gong L. (2015). Comparison of Tripterygium wilfordii Hook F with methotrexate in the treatment of active rheumatoid arthritis (TRIFRA): A randomised, controlled clinical trial. Ann. Rheum. Dis..

[18] Jiang M., Zha Q., Zhang C., Lu C., Yan X., Zhu W., Liu W., Tu S., Hou L., Wang C. (2015). Predicting and verifying outcome of Tripterygium wilfordii Hook F. based therapy in rheumatoid arthritis: From open to double-blinded randomized trial. Sci. Rep..

[19] Jiang Q., Tang X.P., Chen X.C., Xiao H., Liu P., Jiao J. (2017). Will Chinese external therapy with compound Tripterygium wilfordii hook F gel safely control disease activity in patients with rheumatoid arthritis: Design of a double-blinded randomized controlled trial. BMC Complement. Altern. Med..

[20] Wang X., Zu Y., Huang L., Yu J., Zhao H., Wen C., Chen Z., Xu Z. (2017). Treatment of rheumatoid arthritis with combination of methotrexate and Tripterygium wilfordii: A meta-analysis. Life Sci..

[21] Wu C., Jin H.Z., Shu D., Li F., He C.X., Qiao J., Yu X.L., Zhang Y., He Y.B., Liu T.J. (2015). Efficacy and safety of Tripterygium wilfordii hook F versus acitretin in moderate to severe psoriasis vulgaris: A randomized clinical trial. Chin. Med. J..

[22] Liu S., Li X., Li H., Liang Q., Chen J., Chen J. (2015). Comparison of tripterygium wilfordii multiglycosides and tacrolimus in the treatment of idiopathic membranous nephropathy: A prospective cohort study. BMC Nephrol..

[23] Chen Y., Gong Z., Chen X., Tang L., Zhao X., Yuan Q., Cai G. (2013). Tripterygium wilfordii Hook F (a traditional Chinese medicine) for primary nephrotic syndrome. Cochrane Database Syst. Rev..

[24] Ma R.X., Zhao N., Zhang W. (2013). The effects and mechanism of Tripterygium wilfordii Hook F combination with irbesartan on urinary podocyte excretion in diabetic nephropathy patients. Zhonghua Nei Ke Za Zhi.

[25] Ge Y., Xie H., Li S., Jin B., Hou J., Zhang H., Shi M., Liu Z. (2013). Treatment of diabetic nephropathy with Tripterygium wilfordii Hook F extract: A prospective, randomized, controlled clinical trial. J. Transl. Med..

[26] Sun J., Shen X., Dong J., Wang H., Zuo L., Zhao J., Zhu W., Li Y., Gong J., Li J. (2015). Tripterygium wilfordii Hook F as Maintenance Treatment for Crohn’s Disease. Am. J. Med. Sci..

[27] Zhu W., Li Y., Gong J., Zuo L., Zhang W., Cao L., Gu L., Guo Z., Li N., Li J. (2015). Tripterygium wilfordii Hook. f. versus azathioprine for prevention of postoperative recurrence in patients with Crohn’s disease: A randomized clinical trial. Dig. Liver Dis..

[28] Pinna G.F., Fiorucci M., Reimund J.M., Taquet N., Arondel Y., Muller C.D. (2004). Celastrol inhibits pro-inflammatory cytokine secretion in Crohn’s disease biopsies. Biochem. Biophys. Res. Commun..

[29] Fang Z., He D., Yu B., Liu F., Zuo J., Li Y., Lin Q., Zhou X., Wang Q. (2017). High-Throughput Study of the Effects of Celastrol on Activated Fibroblast-Like Synoviocytes from Patients with Rheumatoid Arthritis. Genes.

[30] Xiao S., Zhang M., Liang Y., Wang D. (2017). Celastrol synergizes with oral nifedipine to attenuate hypertension in preeclampsia: A randomized, placebo-controlled, and double blinded trial. J. Am. Soc. Hypertens..

[31] Cleren C., Calingasan N.Y., Chen J., Beal M.F. (2005). Celastrol protects against MPTP- and 3-nitropropionic acid-induced neurotoxicity. J. Neurochem..

[32] Allison A.C., Cacabelos R., Lombardi V.R., Alvarez X.A., Vigo C. (2001). Celastrol, a potent antioxidant and anti-inflammatory drug, as a possible treatment for Alzheimer’s disease. Prog. Neuro Psychopharmacol. Biol. Psychiatry.

[33] Yu J.S., Tseng C.K., Lin C.K., Hsu Y.C., Wu Y.H., Hsieh C.L., Lee J.C. (2017). Celastrol inhibits dengue virus replication via up-regulating type I interferon and downstream interferon-stimulated responses. Antivir. Res..

[34] Kim J.E., Lee M.H., Nam D.H., Song H.K., Kang Y.S., Lee J.E., Kim H.W., Cha J.J., Hyun Y.Y., Han S.Y. (2013). Celastrol, an NF-kappaB inhibitor, improves insulin resistance and attenuates renal injury in db/db mice. PLoS ONE.

[35] Abu Bakar M.H., Cheng K.K., Sarmidi M.R., Yaakob H., Huri H.Z. (2015). Celastrol Protects against Antimycin A-Induced Insulin Resistance in Human Skeletal Muscle Cells. Molecules.

[36] Liu J., Lee J., Salazar Hernandez M.A., Mazitschek R., Ozcan U. (2015). Treatment of obesity with celastrol. Cell.

[37] Lee J.H., Choi K.J., Seo W.D., Jang S.Y., Kim M., Lee B.W., Kim J.Y., Kang S., Park K.H., Lee Y.S. (2011). Enhancement of radiation sensitivity in lung cancer cells by celastrol is mediated by inhibition of Hsp90. Int. J. Mol. Med..

[38] Avilla J., Teixido A., Velazquez C., Alvarenga N., Ferro E., Canela R. (2000). Insecticidal activity of Maytenus species (Celastraceae) nortriterpene quinone methides against codling moth, Cydia pomonella (L.) (Lepidoptera: Tortricidae). J. Agric. Food Chem..

[39] Kim D.H., Shin E.K., Kim Y.H., Lee B.W., Jun J.G., Park J.H., Kim J.K. (2009). Suppression of inflammatory responses by celastrol, a quinone methide triterpenoid isolated from Celastrus regelii. Eur. J. Clin. Investig..

[40] Venkatesha S.H., Yu H., Rajaiah R., Tong L., Moudgil K.D. (2011). Celastrus-derived celastrol suppresses autoimmune arthritis by modulating antigen-induced cellular and humoral effector responses. J. Biol. Chem..

[41] Li H., Yuan Y., Zhang Y., He Q., Xu R., Ge F., Wu C. (2016). Celastrol inhibits IL-1beta-induced inflammation in orbital fibroblasts through the suppression of NF-kappaB activity. Mol. Med. Rep..

[42] Chiang K.C., Tsui K.H., Chung L.C., Yeh C.N., Chen W.T., Chang P.L., Juang H.H. (2014). Celastrol blocks interleukin-6 gene expression via downregulation of NF-kappaB in prostate carcinoma cells. PLoS ONE.

[43] Gao Y., Zhou S., Pang L., Yang J., Li H.J., Huo X., Qian S.Y. (2019). Celastrol suppresses nitric oxide synthases and the angiogenesis pathway in colorectal cancer. Free Radic. Res..

[44] Chen J., Xuan J., Gu Y.T., Shi K.S., Xie J.J., Chen J.X., Zheng Z.M., Chen Y., Chen X.B., Wu Y.S. (2017). Celastrol reduces IL-1beta induced matrix catabolism, oxidative stress and inflammation in human nucleus pulposus cells and attenuates rat intervertebral disc degeneration in vivo. Biomed. Pharmacother. Biomed. Pharmacother..

[45] El-Tanbouly G.S., El-Awady M.S., Megahed N.A., Salem H.A., El-Kashef H.A. (2017). The NF-kappaB inhibitor celastrol attenuates acute hepatic dysfunction induced by cecal ligation and puncture in rats. Environ. Toxicol. Pharmacol..

[46] Han L.P., Li C.J., Sun B., Xie Y., Guan Y., Ma Z.J., Chen L.M. (2016). Protective Effects of Celastrol on Diabetic Liver Injury via TLR4/MyD88/NF-kappaB Signaling Pathway in Type 2 Diabetic Rats. J. Diabetes Res..

[47] Ibuki Y., Mizuno S., Goto R. (2003). gamma-Irradiation-induced DNA damage enhances NO production via NF-kappaB activation in RAW264.7 cells. Biochim. Biophys. Acta.

[48] Manna K., Das U., Das D., Kesh S.B., Khan A., Chakraborty A., Dey S. (2015). Naringin inhibits gamma radiation-induced oxidative DNA damage and inflammation, by modulating p53 and NF-kappaB signaling pathways in murine splenocytes. Free Radic. Res..

[49] Li F., Zhang J., Arfuso F., Chinnathambi A., Zayed M.E., Alharbi S.A., Kumar A.P., Ahn K.S., Sethi G. (2015). NF-kappaB in cancer therapy. Arch. Toxicol..

[50] Li F., Sethi G. (2010). Targeting transcription factor NF-kappaB to overcome chemoresistance and radioresistance in cancer therapy. Biochim. Biophys. Acta.

[51] Ahn K.S., Sethi G., Chaturvedi M.M., Aggarwal B.B. (2008). Simvastatin, 3-hydroxy-3-methylglutaryl coenzyme A reductase inhibitor, suppresses osteoclastogenesis induced by receptor activator of nuclear factor-kappaB ligand through modulation of NF-kappaB pathway. Int. J. Cancer.

[52] Nair A.S., Shishodia S., Ahn K.S., Kunnumakkara A.B., Sethi G., Aggarwal B.B. (2006). Deguelin, an Akt inhibitor, suppresses IkappaBalpha kinase activation leading to suppression of NF-kappaB-regulated gene expression, potentiation of apoptosis, and inhibition of cellular invasion. J. Immunol..

[53] Ahn K.S., Sethi G., Jain A.K., Jaiswal A.K., Aggarwal B.B. (2006). Genetic deletion of NAD(P)H:quinone oxidoreductase 1 abrogates activation of nuclear factor-kappaB, IkappaBalpha kinase, c-Jun N-terminal kinase, Akt, p38, and p44/42 mitogen-activated protein kinases and potentiates apoptosis. J. Biol. Chem..

[54] Manna S.K., Aggarwal R.S., Sethi G., Aggarwal B.B., Ramesh G.T. (2007). Morin (3,5,7,2′,4′-Pentahydroxyflavone) abolishes nuclear factor-kappaB activation induced by various carcinogens and inflammatory stimuli, leading to suppression of nuclear factor-kappaB-regulated gene expression and up-regulation of apoptosis. Clin. Cancer Res..

[55] Ahn K.S., Sethi G., Aggarwal B.B. (2008). Reversal of chemoresistance and enhancement of apoptosis by statins through down-regulation of the NF-kappaB pathway. Biochem. Pharmacol..

[56] Chua A.W., Hay H.S., Rajendran P., Shanmugam M.K., Li F., Bist P., Koay E.S., Lim L.H., Kumar A.P., Sethi G. (2010). Butein downregulates chemokine receptor CXCR4 expression and function through suppression of NF-kappaB activation in breast and pancreatic tumor cells. Biochem. Pharmacol..

[57] Siveen K.S., Mustafa N., Li F., Kannaiyan R., Ahn K.S., Kumar A.P., Chng W.J., Sethi G. (2014). Thymoquinone overcomes chemoresistance and enhances the anticancer effects of bortezomib through abrogation of NF-kappaB regulated gene products in multiple myeloma xenograft mouse model. Oncotarget.

[58] Manu K.A., Shanmugam M.K., Li F., Chen L., Siveen K.S., Ahn K.S., Kumar A.P., Sethi G. (2014). Simvastatin sensitizes human gastric cancer xenograft in nude mice to capecitabine by suppressing nuclear factor-kappa B-regulated gene products. J. Mol. Med..

[59] Pfalzgraff A., Heinbockel L., Su Q., Gutsmann T., Brandenburg K., Weindl G. (2016). Synthetic antimicrobial and LPS-neutralising peptides suppress inflammatory and immune responses in skin cells and promote keratinocyte migration. Sci. Rep..

[60] Kang K., Won M., Yuk J.M., Park C.Y., Byun H.S., Park K.A., Lee S.R., Kang Y.G., Shen H.M., Lee I.Y. (2016). IinQ attenuates systemic inflammatory responses via selectively impairing the Myddosome complex formation upon TLR4 ligation. Biochem. Pharmacol..

[61] Dong X.R., Luo M., Fan L., Zhang T., Liu L., Dong J.H., Wu G. (2010). Corilagin inhibits the double strand break-triggered NF-kappaB pathway in irradiated microglial cells. Int. J. Mol. Med..

[62] Wang H., Sim M.K., Loke W.K., Chinnathambi A., Alharbi S.A., Tang F.R., Sethi G. (2017). Potential Protective Effects of Ursolic Acid against Gamma Irradiation-Induced Damage Are Mediated through the Modulation of Diverse Inflammatory Mediators. Front. Pharmacol..

[63] Kuntic V.S., Stankovic M.B., Vujic Z.B., Brboric J.S., Uskokovic-Markovic S.M. (2013). Radioprotectors—The evergreen topic. Chem. Biodivers..

[64] Sandeep D., Nair C.K. (2011). Radioprotection by alpha-asarone: Prevention of genotoxicity and hematopoietic injury in mammalian organism. Mutat. Res..

[65] Ran Y., Wang R., Hasan M., Jia Q., Tang B., Shan S., Deng Y., Qing H. (2014). Radioprotective effects of dragon’s blood and its extracts on radiation-induced myelosuppressive mice. J. Ethnopharmacol..

[66] Zheng H., Wang S., Zhou P., Liu W., Ni F. (2013). Effects of Ligustrazine on DNA damage and apoptosis induced by irradiation. Environ. Toxicol. Pharmacol..

[67] Luo Q., Cui X., Yan J., Yang M., Liu J., Jiang Y., Li J., Zhou Y. (2011). Antagonistic effects of Lycium barbarum polysaccharides on the impaired reproductive system of male rats induced by local subchronic exposure to 60Co-gamma irradiation. Phytother. Res..

[68] Lata M., Prasad J., Singh S., Kumar R., Singh L., Chaudhary P., Arora R., Chawla R., Tyagi S., Soni N.L. (2009). Whole body protection against lethal ionizing radiation in mice by REC-2001: A semi-purified fraction of Podophyllum hexandrum. Phytomedicine.

[69] Sankhwar S., Gupta M.L., Gupta V., Verma S., Suri K.A., Devi M., Sharma P., Khan E.A., Alam M.S. (2011). Podophyllum hexandrum-Mediated Survival Protection and Restoration of Other Cellular Injuries in Lethally Irradiated Mice. Evid. Based Complement. Altern. Med..

[70] Dutta A., Verma S., Sankhwar S., Flora S.J., Gupta M.L. (2012). Bioavailability, antioxidant and non toxic properties of a radioprotective formulation prepared from isolated compounds of Podophyllum hexandrum: A study in mouse model. Cell. Mol. Biol..

[71] Saini R., Verma S., Singh A., Lata Gupta M. (2013). Role of Active Principles of Podophyllum hexandrum in Amelioration of Radiation Mediated Lung Injuries by Reactive Oxygen/Nitrogen Species Reduction. CellBio.

[72] Chaudhary P., Shukla S.K., Sharma R.K. (2011). REC-2006-A Fractionated Extract of Podophyllum hexandrum Protects Cellular DNA from Radiation-Induced Damage by Reducing the Initial Damage and Enhancing Its Repair In Vivo. Evid. Based Complement. Altern. Med..

[73] Sandeep D., Nair C.K. (2010). Protection of DNA and membrane from gamma-radiation induced damage by the extract of Acorus calamus Linn.: An in vitro study. Environ. Toxicol. Pharmacol..

[74] Sandeep D., Nair C.K. (2012). Protection from lethal and sub-lethal whole body exposures of mice to gamma-radiation by Acorus calamus L.: Studies on tissue antioxidant status and cellular DNA damage. Exp. Toxicol. Pathol..

[75] El-Desouky W., Hanafi A., Abbas M.M. (2016). Radioprotective effect of green tea and grape seed extracts mixture on gamma irradiation induced immune suppression in male albino rats. Int. J. Radiat. Biol..

[76] Ismail A.F., Salem A.A., Eassawy M.M. (2016). Modulation of gamma-irradiation and carbon tetrachloride induced oxidative stress in the brain of female rats by flaxseed oil. J. Photochem. Photobiol..

[77] Dai Y., DeSano J.T., Meng Y., Ji Q., Ljungman M., Lawrence T.S., Xu L. (2009). Celastrol potentiates radiotherapy by impairment of DNA damage processing in human prostate cancer. Int. J. Radiat. Oncol. Biol. Phys..

[78] Stankova K., Ivanova K., Nikolov V., Aneva N., Georgieva R., Boteva R. (2013). Proteasome inhibition protects human peripheral blood mononuclear cells from radiation-induced oxidative stress. Int. J. Radiat. Biol..

[79] Han X.B., Tan Y., Fang Y.Q., Li F. (2018). Protective effects of celastrol against gamma irradiation-induced oxidative stress in human umbilical vein endothelial cells. Exp. Ther. Med..

[80] Yu H., Aravindan N., Xu J., Natarajan M. (2017). Inter- and Intra-cellular Mechanism of NF-kB-dependent Survival Advantage and Clonal Expansion of Radio-resistant Cancer Cells. Cell. Signal..

[81] Lee Y.H., Wang E., Kumar N., Glickman R.D. (2014). Ursolic acid differentially modulates apoptosis in skin melanoma and retinal pigment epithelial cells exposed to UV-VIS broadband radiation. Apoptosis.

[82] Das U., Manna K., Sinha M., Datta S., Das D.K., Chakraborty A., Ghosh M., Saha K.D., Dey S. (2014). Role of ferulic acid in the amelioration of ionizing radiation induced inflammation: A murine model. PLoS ONE.

[83] Khan S., Adhikari J.S., Rizvi M.A., Chaudhury N.K. (2015). Radioprotective potential of melatonin against ^60^Co gamma-ray-induced testicular injury in male C57BL/6 mice. J. Biomed. Sci..

[84] Kindekov I., Mileva M., Krastev D., Vassilieva V., Raynova Y., Doumanova L., Aljakov M., Idakieva K. (2014). Radioprotective effect of Rapana thomasiana hemocyanin in gamma induced acute radiation syndrome. Biotechnol. Biotechnol. Equip..

[85] Hu J., Yang Z., Wang J., Tang Y., Liu H., Zhang B., Chen H. (2013). Infusion of Trx-1-overexpressing hucMSC prolongs the survival of acutely irradiated NOD/SCID mice by decreasing excessive inflammatory injury. PLoS ONE.

[86] Mortazavi S.M., Rahimi S., Mosleh-Shirazi M.A., Arjomandi M., Soleimani A., Koohi Hossein-Abadi O., Haghani M., Alavi M. (2015). A Comparative Study on the Life-Saving Radioprotective Effects of Vitamins A, E, C and Over-the-Counter Multivitamins. J. Biomed. Phys. Eng..

[87] Booth C., Tudor G., Tudor J., Katz B.P., MacVittie T.J. (2012). Acute gastrointestinal syndrome in high-dose irradiated mice. Health Phys..

[88] Rosen E.M., Day R., Singh V.K. (2014). New approaches to radiation protection. Front. Oncol..

[89] Williams J.P., Brown S.L., Georges G.E., Hauer-Jensen M., Hill R.P., Huser A.K., Kirsch D.G., Macvittie T.J., Mason K.A., Medhora M.M. (2010). Animal models for medical countermeasures to radiation exposure. Radiat. Res..

[90] Kusy S., Ghosn E.E., Herzenberg L.A., Contag C.H. (2012). Development of B cells and erythrocytes is specifically impaired by the drug celastrol in mice. PLoS ONE.

[91] Venkatesha S.H., Dudics S., Astry B., Moudgil K.D. (2016). Control of autoimmune inflammation by celastrol, a natural triterpenoid. Pathog. Dis..

[92] Zhang J., Shan J., Chen X., Li S., Long D., Li Y. (2018). Celastrol mediates Th17 and Treg cell generation via metabolic signaling. Biochem. Biophys. Res. Commun..

[93] Astry B., Venkatesha S.H., Laurence A., Christensen-Quick A., Garzino-Demo A., Frieman M.B., O’Shea J.J., Moudgil K.D. (2015). Celastrol, a Chinese herbal compound, controls autoimmune inflammation by altering the balance of pathogenic and regulatory T cells in the target organ. Clin. Immunol..

[94] Abdin A.A., Hasby E.A. (2014). Modulatory effect of celastrol on Th1/Th2 cytokines profile, TLR2 and CD3+ T-lymphocyte expression in a relapsing-remitting model of multiple sclerosis in rats. Eur. J. Pharmacol..

[95] Sinha M., Das D.K., Manna K., Datta S., Ray T., Sil A.K., Dey S. (2012). Epicatechin ameliorates ionising radiation-induced oxidative stress in mouse liver. Free Radic. Res..

[96] Brach M.A., Hass R., Sherman M.L., Gunji H., Weichselbaum R., Kufe D. (1991). Ionizing radiation induces expression and binding activity of the nuclear factor kappa B. J. Clin. Investig..

